# Soil chemistry and microbiome modulation through water irrigation containing oxygen, hydrogen, and carbon dioxide nanobubbles

**DOI:** 10.1128/aem.02173-25

**Published:** 2026-03-30

**Authors:** Nguyen Nhat Thu Le, Shan Xue, Hui Mu, Jianfeng Wu, Chuanwu Xi, Taha Marhaba, Wen Zhang

**Affiliations:** 1 Department of Environmental Health Sciences, University of Michigan School of Public Health 51329, Ann Arbor, Michigan, USA; 2 Department of Civil and Environmental Engineering, New Jersey Institute of Technology 5965, Newark, New Jersey, USA; 3 PureNanoTech Inc., Parsippany, New Jersey, USA; 4 School of Water Conservancy and Environment, University of Jinan 12413, Jinan, China; University of Nebraska-Lincoln, Lincoln, Nebraska, USA

**Keywords:** nanobubbles, soil microbiome, metabolic pathways prediction, keystone taxa, sustainable agriculture

## Abstract

Nanobubble technology has shown considerable promise in sustainable agriculture due to its ability to enhance seed germination, plant growth, and soil quality without leaving harmful residues. While benefits are often attributed to improved soil aeration and soil texture changes (e.g., permeability), nanobubbles may also influence soil microbiome, an essential driver of plant health and nutrient cycling. This study systematically investigated how different nanobubble types—oxygen, hydrogen, and carbon dioxide—modulate soil chemistry and microbial community structure over a 4-week period. Multivariate analyses on microbiome taxonomic composition revealed distinct microbial responses to each gas type. Oxygen and hydrogen nanobubble treatments resulted in more pronounced shifts in microbial composition and functional potential compared to carbon dioxide nanobubbles. These shifts included enrichment of bacterial taxa associated with nutrient turnover, pollutant degradation, and pathogen suppression, such as *Flavobacteriaceae*, *Comamonadaceae*, *Nannocystaceae*, and *Blastocatellaceae*. Functional predictions showed that oxygen and hydrogen nanobubbles could promote metabolic pathways related to organic compound degradation and remediation of contaminated soil. Microbial network analysis further highlighted the beneficial impacts of nanobubbles on keystone taxa, such as *Flavobacteriaceae*, which in turn play pivotal roles in shaping soil ecosystem functions. Together, these findings demonstrate that gas-specific nanobubble irrigation can steer soil microbiome dynamics in ways that may enhance soil fertility, resilience, and crop productivity.

**IMPORTANCE:**

This study provides new insights into how nanobubble irrigation can be used to improve soil health and agricultural sustainability. By demonstrating that oxygen and hydrogen nanobubbles selectively enrich beneficial microbial taxa linked to soil nutrient turnover, pollution degradation, and pathogen suppression, this study identifies a promising approach to enhance plant growth and soil health through new nanobubble-driven processes. The detection of keystone taxa responsive to nanobubble treatments also reveals potential microbial mechanisms underlying the interactions between nanobubbles, soil, and plant health. Together, these findings highlight nanobubble irrigation as a novel and scalable strategy for microbiome engineering that could advance sustainable crop production and environmental stewardship. Furthermore, while prior studies have primarily focused on the microbial effects of air and oxygen nanobubbles, our study systematically examined and compared the impacts of less explored nanobubble types, specifically hydrogen and carbon dioxide, demonstrating the broad versatility of nanobubbles for diverse agricultural applications.

## INTRODUCTION

Nanobubbles are ultrafine gaseous bubbles in water, typically with a diameter of around 100 to 200 nm (1, 2). Owing to their nanoscale sizes and high internal pressures, nanobubbles exhibit unusual physicochemical properties, including long-term stability (3–5), high gas solubility (6), and the ability to generate reactive oxygen species (ROS) when they collapse (7). In agricultural applications, nanobubble-infused water irrigation has repeatedly been shown to promote seed germination (7–9), plant growth (8, 9), and crop yield (8, 10–12). As a result, the use of nanobubble water in crop production can potentially reduce irrigation demand and chemical fertilizer input, which in turn can mitigate water scarcity and water pollution (e.g., eutrophication) or soil erosion caused by excessive irrigation or fertilizer use (10, 13).

The beneficial impacts of nanobubbles on plant growth are mediated via multiple proven and speculated mechanisms. For instance, air and oxygen nanobubbles improve soil oxygen availability and enhance root respiration (10, 12, 14, 15). The ROS generated by nanobubbles can function as cell-wall loosening agents and essential signaling molecules, stimulating plant cell proliferation and survival when maintained at moderate ROS levels (7, 16, 17). In addition, the charged surfaces of nanobubbles promote the release of nutrient ions from soil and increase their bioavailability to plants (13, 18). Beyond these direct effects, nanobubbles may also exert indirect effects by modulating soil microbial communities, which play key roles in nutrient cycling, organic matter degradation, and plant pathogen suppression (19, 20). Many soil and rhizosphere bacteria also produce hormones, such as auxins, cytokinins, and gibberellins, which could stimulate plant root development and enhance plant nutrient uptake (21, 22). Soil and rhizosphere microbiomes are highly responsive to shifts in environmental parameters, such as redox potential, oxygen levels, and pH, all of which can be altered by nanobubbles (23, 24). Oxygen nanobubbles were reported to enhance the abundance of aerobic and nitrogen-cycling microorganisms (11, 25). Chen et al. also reported that oxygen nanobubbles increase soil microbial diversity and reduce biofilm formation, thereby improving soil permeability and tomato yield (26). In addition, oxygen and nitrogen nanobubble treatments have been shown to promote bacteria involved in organic matter degradation and enhance microbial interactions in the tomato rhizosphere microbiome (27). These findings highlight that the soil microbiome acts as a key ecological mediator linking nanobubble exposure to improved soil quality and plant performance.

However, existing studies have largely focused on the impacts of air or oxygen nanobubbles (26, 28–30), with limited understanding of how other gas types (e.g., nitrogen, hydrogen, or carbon dioxide) modulate the soil microbes and influence soil health. Recent studies have shown that carbon dioxide nanobubbles can improve seed germination and seedling growth in several crops, such as bean, carrot, and tomato (9, 31). Hydrogen nanobubbles have been reported to increase tomato yield and antioxidant content (32, 33). Given the distinct chemical properties of these gases, their effects on microbial taxa, community diversity, and functional pathways may vary substantially. A comparative evaluation of the effects of gas-specific nanobubbles on soil microbial communities is therefore crucial to elucidate the mechanisms underlying nanobubble-based plant growth promotion and to guide precision agricultural applications.

The aims of his study included (i) a systematic comparison of the soil microbial responses to nanobubbles with different gas cores (CO_2_, H_2_, and O_2_) over a 4-week treatment period; (ii) a multi-level characterization of soil microbial diversity and community structure under different treatments in terms of alpha and beta diversity, taxonomic composition, predicted metabolic functions, and co-occurrence network; and (iii) examination of the possible mechanisms linking nanobubble-induced soil physicochemical changes and microbiome shifts. By identifying both shared and treatment-specific microbial responses, this work provides new mechanistic insights into nanobubble–soil interactions and highlights the potential to tailor nanobubble technology for diverse agricultural contexts.

## MATERIALS AND METHODS

### Nanobubble water preparation and characterization

Nanobubbles containing either CO_2_, H_2_, or O_2_ gas were generated in DI water using our reported membrane bubbling technique (27, 34). Briefly, compressed CO_2_, H_2_, or O_2_ gas was injected into 500 mL DI water through a commercial nanobubble generator (Purenano Tech. Inc., USA) with a gas flow rate of 0.45 L·m^−1^ under a pressure of 60 psi. The produced nanobubbles of three kinds of gases exhibited a similar bubble size distribution of 100–200 nm and concentration of 4 ± 0.8 × 10^8^ bubbles·mL^−1^ (35). CO_2_ nanobubbles rendered a zeta potential of −20 to −30 mV, while H_2_ and O_2_ nanobubbles had −30 to −45 mV in DI water, as measured by dynamic light scattering using a NanoSight NS300 instrument (Malvern Panalytical, UK) and a Zetasizer Nano ZS instrument (Malvern Panalytical, UK).

### Soil preparation and treatment with nanobubble water

Miracle-Gro potting soil (OMS Investments Inc., USA) was sieved using a U.S. #10 mesh to remove particles larger than 2 mm in diameter before use. The soil had a pH value of 7.00 ± 0.35, electrical conductivity of 8,583.1 ± 35.6 μs·cm^−1^, total organic carbon of 170.7 ± 8.5 g·kg^−1^, total nitrogen of 13.5 ± 0.2 g·kg^−1^, total phosphorus of 0.21 ± 0.01 g·kg^−1^, and organic matter content of 21.4% ± 1.1 %. Soil columns were made by adding 4 g of soil into 50-mL polypropylene centrifuge tubes, as shown in Fig. S1 in the Supporting Information. The packing density was approximately 1 g·cm^−3^, which is close to the typical compactness of agricultural soil (36). Each tube had a 0.5-cm hole at the bottom for water drainage, with six glass beads (6 mm in diameter) placed at the bottom to support the soil column and prevent soil loss.

Every day, 5 mL of the nanobubble water (containing CO_2_, H_2_, or O_2_) or DI water (control) was sprayed onto the soil surface for four consecutive weeks, which resulted in a variation of soil moisture content shift from the original 25% to 40% every day. Also, the original soil, which was not exposed to either nanobubbles or DI water, served as the negative control group. To minimize evaporation, the tubes were covered with caps with a 0.3-cm vent hole. The dissolved oxygen (DO), pH, and redox potential of the moist soil were recorded every day following nanobubble water treatment. A total of 20 soil columns were prepared for each treatment group. Every week, five columns were collected for microbial DNA extraction and sequencing. Samples were preserved in a 12-mL DESS solution (20% dimethyl sulfoxide, 0.25 M disodium EDTA, and 6.14 M NaCl) (37, 38) during transport to the University of Michigan for DNA extraction and 16S rRNA gene sequencing.

### Bacterial DNA extraction and 16S rRNA gene sequencing

Total soil DNA was extracted using the RNeasy PowerSoil DNA Elution Kit (Qiagen, USA). The V4 region of the bacterial 16S rRNA gene was amplified using the primer pair 515F (5′-GTGCCAGCMGCCGCGGTAA-3′) and 806R (5′-GGACTACHVGGGTWTCTAAT-3′). Amplicon sequencing was performed on the Illumina MiSeq platform at the University of Michigan Microbiome Core using a paired-end (250 bp × 2) protocol developed by Kozich et al. (39). The standard operating procedure (SOP) for this sequencing workflow is available at https://github.com/SchlossLab/MiSeq_WetLab_SOP.

### Microbial community analysis

Details of the bioinformatic pipelines and parameters are provided in Text S1 of the Supporting Information. Briefly, the raw DNA sequences were filtered, merged, and denoised using DADA2 (v. 1.32.0) in R version 4.4.1 (40). The filtered sequences were then aligned to the SILVA database (v. 138.1) to obtain an amplicon sequence variant (ASV) table and rarefied to 2,120 reads per sample. Rarefaction curves show that the numbers of ASVs were near the plateau at this depth for all samples (Fig. S2).

Microbial community analyses were carried out in R version 4.4.1. Alpha diversity was measured in terms of observed richness and Shannon index, while beta diversity was assessed with Adonis tests based on Bray-Curtis dissimilarity (41). The effect of increasing duration of nanobubble exposure on microbial community structure was examined by canonical correspondence analysis (CCA). Differentially abundant bacterial taxa between treatment groups were identified using the linear discriminant analysis effect size (LEfSe) method (42). The functional profiles of the soil microbiome samples were predicted using the Phylogenetic Investigation of Communities by Reconstruction of Unobserved States (PICRUSt2) pipeline v2.3.2 (43). Microbial co-occurrence network analysis was carried out at the family level using the NetCoMi package in R (44).

### Statistical analysis

The Kruskal-Wallis test and the Conover-Iman post hoc test with a significant level α = 0.05 were used to identify significant changes in the soil DO levels, redox potentials, pH values, and alpha diversity metrics under different nanobubble types and treatment durations compared to the control. The Benjamini-Hochberg procedure was used in the post hoc test to adjust for false discoveries. Statistical tests were performed using R version 4.4.1.

## RESULTS AND DISCUSSION

### Effects of different nanobubbles on soil oxygen level, pH, and redox potential

**Fig 1:**
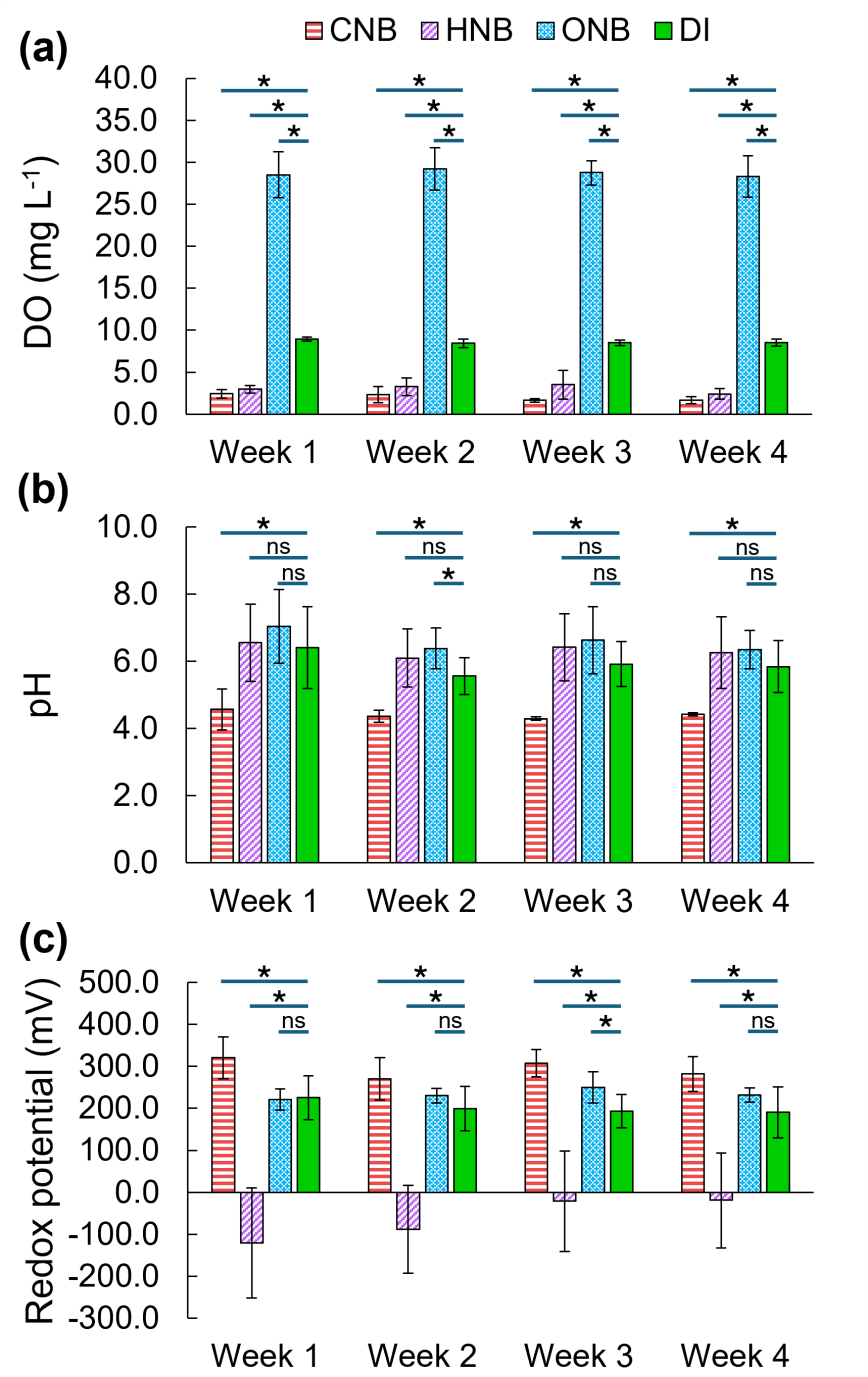
Soil characteristics under irrigation with water containing CO_2_ nanobubbles (CNB), H_2_ nanobubbles (HNB), O_2_ nanobubbles (ONB), and DI water over 4 weeks: (**a**) dissolved oxygen level, (**b**) pH, and (**c**) redox potential. Error bars represent standard deviations. Asterisks (*) indicate statistically significant differences based on Kruskal-Wallis and Conover tests, while “ns” indicates no statistically significant difference.

Figure 1 compares the changes in the soil oxygen levels, pH, and redox potentials after irrigation with water containing carbon dioxide nanobubbles (CNBs), hydrogen nanobubbles (HNBs), oxygen nanobubbles (ONBs), or DI water. It was observed that ONBs significantly increased soil oxygen level, reaching an average of 28.7 mg·L^−1^, compared to 8.6 mg·L^−1^ in the DI control (Fig. 1a). By contrast, CNBs and HNBs significantly decreased soil DO level to 2.0 and 3.0 mg·L^−1^, respectively, which agrees with previous studies on the impacts of nanobubbles containing carbon dioxide, hydrogen, and nitrogen on soil oxygen content (9, 18). In addition, HNBs have been reported to increase the microbial oxygen consumption in soil, leading to reduced DO in the soil pore water (45).

For soil pH impacts, CNBs significantly reduced soil pH to about 4.4, while other treatments remained between 5.9 and 6.6 (Fig. 1b). This was caused by the reaction of CO_2_ gas released from CNBs with water to form carbonic acid (H_2_CO_3_) (46). In contrast, no statistically significant differences were observed between HNB- and ONB-treated soils and the control.

Distinct patterns were observed in redox potential responses across treatments (Fig. 1c). CNB-treated soil was significantly more oxidized (295 mV on average) than control (202 mV on average), while ONB-treated soil only had significantly higher redox potential than control in week 3 and no significant difference in other weeks. The increased redox potential of CNB-treated soil can be explained by CO_2_-induced acidification (47). According to the Nernst equation (Text S2), a decrease in pH tends to increase the redox potential. In contrast, HNB-treated soil had the lowest redox potential, ranging from −121 to −19 mV from weeks 1 to 4. Hydrogen can act as an electron donor for reductive microbial processes, such as methanogenesis, denitrification, and sulfate reduction (48, 49), accounting for the low redox potential of HNB-treated soil. Notably, the redox potential of HNB-treated soil increased gradually over time during the treatment period. This trend likely reflects microbial community adaptation to HNBs, leading to a higher rate of hydrogen consumption, which reduced the strength of reductive processes in later weeks (23, 50). Certain microbial taxa are also known to raise soil redox potential, such as manganese-oxidizing bacteria (51) and *Micrococcus luteus* (52). The *P* values for the Kruskal-Wallis tests and post hoc tests comparing soil parameters under different nanobubble treatments are shown in Table S1.

These findings highlight that nanobubble gases exert distinct effects on soil geochemical conditions: ONBs strongly enhanced oxygen availability, CNBs acidified soil and elevated redox potential despite lower DO, and HNBs drove soils to highly reduced states before a gradual recovery. Such shifts in DO, pH, and redox potential are expected to have strong downstream effects on microbial community structure and function.

### Nanobubble-induced shifts in soil bacterial diversity and community composition

#### Temporal trends in soil microbial diversity

**Fig 2:**
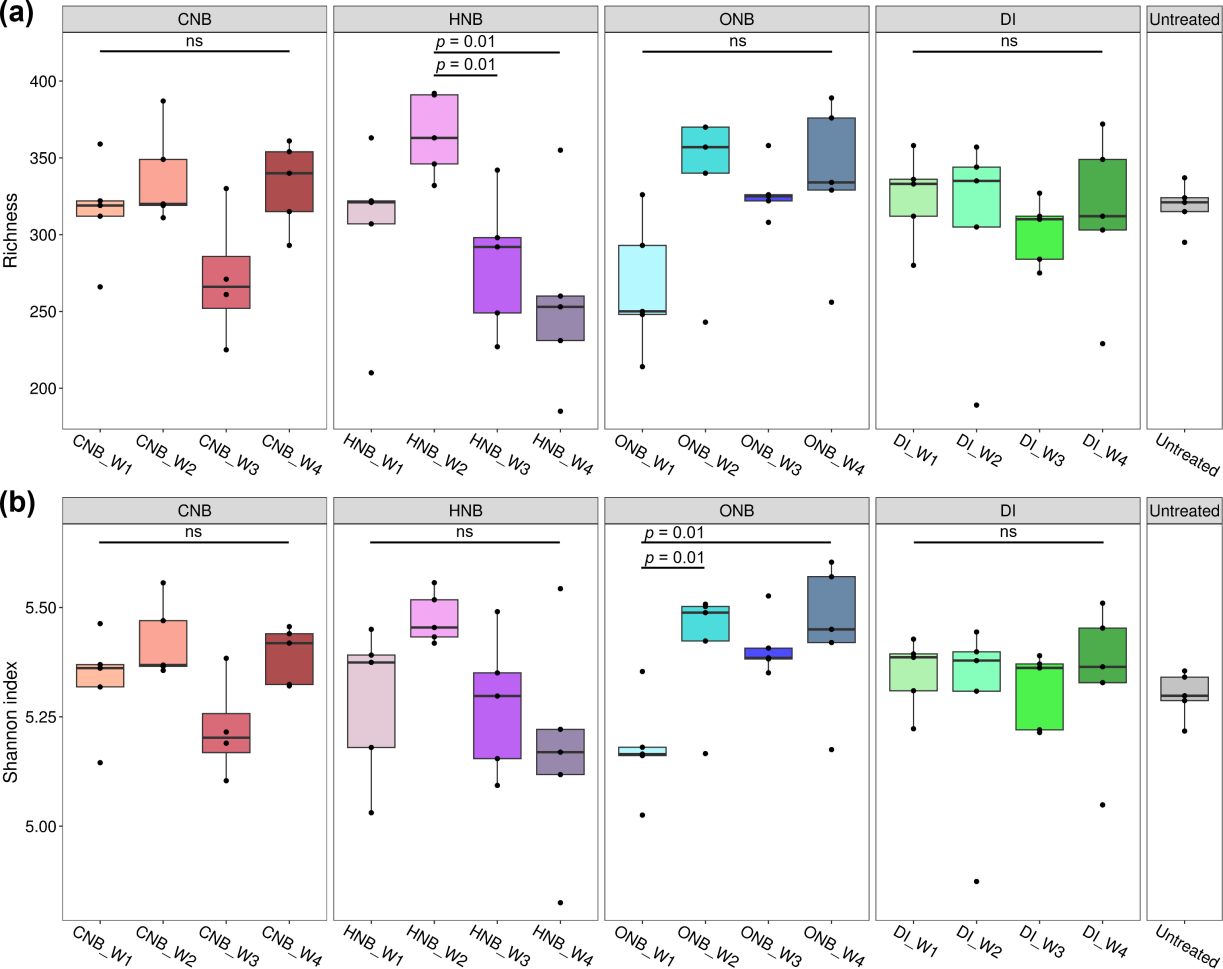
Alpha diversity metrics including (**a**) observed richness and (**b**) Shannon index of soil microbial communities under different irrigation treatments: CNB, HNB, ONB, or DI water. Samples were collected weekly from week 1 (W1) to week 4 (W4). The untreated soil is included for comparison.

The effects of different nanobubble types on soil microbial diversity were evaluated using species richness and Shannon index. Species richness represents the number of observed ASVs, while the Shannon index incorporates both richness and evenness, thus capturing the distribution of species within the community (53). Higher values of these indices indicate greater diversity and potentially more stable microbial communities (54). Figure 2 shows that overall, nanobubble-treated samples did not have significantly different alpha diversity metrics compared to DI water-treated samples in any week. Nevertheless, distinct temporal trends emerged among different gas types. Soils treated with CNBs exhibited fluctuations in both richness and Shannon index. This outcome may be linked to CO_2_-induced acidification, which can suppress certain microbial groups. However, the short treatment duration (1 month) was likely insufficient to substantially alter overall diversity. HNB treatment resulted in an initial increase in richness and Shannon index between weeks 1 and 2, followed by a significant decline in richness during weeks 3 and 4 (Conover *P* = 0.01; Table S1). Since this pattern mirrors the changes in redox potential of HNB-treated soil, the peak in diversity likely corresponded to a proliferation of hydrogen-oxidizing bacteria that thrive in the strongly reductive conditions in the first 2 weeks of HNB treatment, whereas the subsequent decrease in diversity reflects the inhibition of bacterial taxa that preferred more oxidized conditions.

In contrast, ONB-treated soils showed relatively low α diversity in week 1, followed by a gradual increase in subsequent weeks. The Shannon index of ONB-treated soils in weeks 2 and 4 was significantly higher than that in week 1 (Conover *P* = 0.01; Table S1). This trend may be attributed to the aeration effect of ONBs, which reduced the abundance of anaerobic taxa in the short term while stimulating aerobic and facultative microorganisms in the long term, thereby increasing community diversity. Previous studies have generally reported increased diversity under oxygen or air nanobubble irrigation (26, 55, 56), although opposite outcomes, where alpha diversity decreased following ONB treatment, have also been documented (30, 57). These discrepancies suggest that the impact of ONBs on microbial diversity may depend strongly on initial community structure and soil physicochemical conditions. For instance, soils dominated by anaerobes may exhibit lower microbial diversity upon exposure to ONBs, while soils containing a mixture of aerobic, anaerobic, and facultative taxa may gain diversity.

The sequencing depth after rarefaction was relatively low (2,120 reads per sample), which likely reduced statistical power and led to an underestimation of alpha diversity. This limitation may explain the lack of observed differences in microbial diversity between treatment and control groups. In addition, the treatment duration (1 month) was relatively short, as the study was designed to capture the immediate effects of nanobubble irrigation and detailed microbial responses over time. Notably, ONB-treated soils exhibited a trend toward increasing diversity over time, whereas DI water-treated soils showed no temporal trends. These results suggest that longer ONB treatment durations may elicit statistically significant increases in microbial diversity, warranting further investigation in future studies.

#### Phylum-level microbial shifts in response to nanobubbles

The top 20 phyla in the soil microbiome samples and their relative abundances are shown in Fig. S3. The top five phyla were *Proteobacteria* (21.2%), *Bacteroidota* (17.8%), *Chloroflexi* (11.6%), *Acidobacteriota* (5.6%), and *Planctomycetota* (3.8%). Irrigation duration had a stronger impact on the soil microbiome composition at the phylum level than nanobubble type. All nanobubble-treated groups and control (DI water) showed gradual increases in the relative abundances of *Acidobacteriota* and *Firmicutes* from week 1 to week 4, while the relative abundances of *Bacteroidota* and *Nanoarchaeota* gradually decreased. Differences in microbial composition between the nanobubble-treated groups and the control were not apparent at the phylum level.

#### Changes in microbial community composition

**Fig 3:**
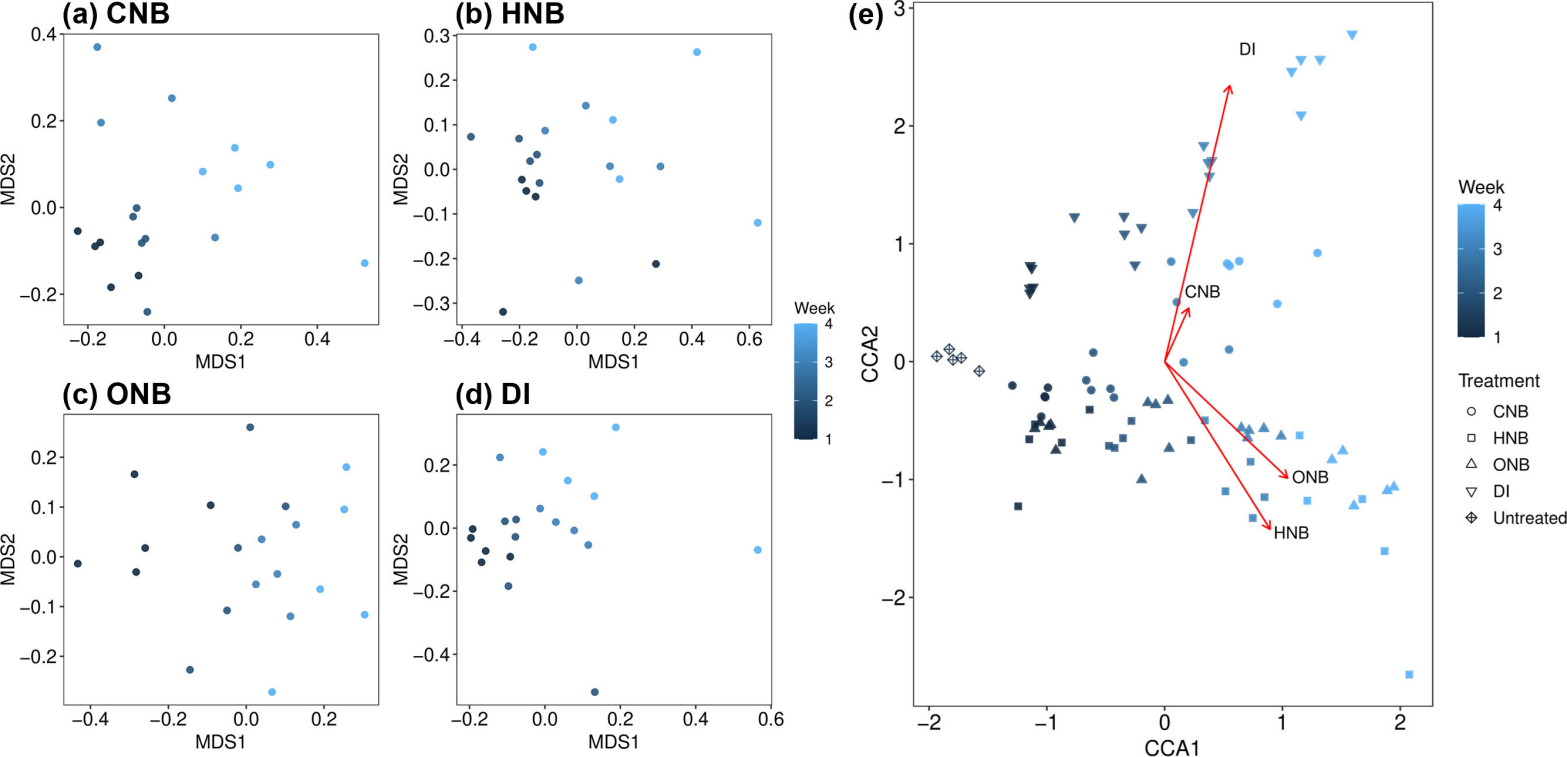
(**a–d**) NMDS plots showing soil microbiome structures under different treatments: CNB, HNB, ONB, or control (DI) over 4 weeks. Samples were collected at the end of each week. (**e**) CCA plot showing the extent and direction of the effects exerted by irrigation water type (CNB, HNB, ONB, or DI) on soil microbiome structure. Untreated soil microbiome is also included for comparison.

Non-metric multidimensional scaling (NMDS) and Adonis tests were used to compare the overall microbial community structures under different irrigation treatments. The NMDS plots in Fig. 3a through d show gradual shifts in soil microbiome composition with increasing exposure time to each nanobubble treatment or DI water. ONB-treated microbiomes exhibited the most pronounced changes from one week to the next, as shown in Fig. 3c. Adonis tests further confirm that the ONB-treated microbiomes from all four sampling times had significantly different compositions (Table S2). While the week-to-week changes in CNB- and HNB-treated microbiomes were less consistent than in ONB-treated samples, CNB- and HNB-treated microbiomes still displayed significant shifts from weeks 1–2 to weeks 3–4, as shown by NMDS plots (Fig. 3a and b) and Adonist test results (Table S2).

Several studies have reported significant alterations in the soil microbiome structure following air or oxygen nanobubble treatment (26, 30, 55, 56). Zhou et al. in particular reported that the rhizosphere soil microbiome of tomato plants differentiated with the increasing ONB concentration (30). Our results complement existing studies by showing that the effects of CO_2_, H_2_, and O_2_ nanobubbles on soil microorganisms also accumulate over increasing treatment duration, thus providing evidence that long-term irrigation with nanobubble water can have lasting impacts on the soil microbiome. Regarding the effects of nanobubble type, ONB-treated microbiomes and HNB-treated microbiomes tended to have similar community structures, but both were significantly different from the DI control at each sampling time, as indicated by Adonis test results (*P* < 0.05, Table S3). Meanwhile, CNB-treated microbiomes were significantly different from the control in week 1 (*P* = 0.007) and week 2 (*P* = 0.009) but became more similar to the control in week 3 (*P* = 0.054) and week 4 (*P* = 0.073). In week 4, CNB-treated samples were also significantly different from HNB- and ONB-treated samples (*P* = 0.026 and *P* = 0.009, respectively). An NMDS plot with samples from all three nanobubble treatments, DI control, and the untreated soil control can be found in Fig. S4.

CCA was also used to examine how increasing nanobubble treatment duration affected the soil microbiome structure. As shown in Fig. 3e, HNBs and ONBs altered soil microbiome structure in similar directions, while CNBs shifted soil microbiome structure in a similar direction as DI water treatment. All four variables (CNB, HNB, ONB, and DI exposure duration) were statistically significant in the CCA model (*P* = 0.001 for each variable).

### Microbial taxa enriched under nanobubble irrigation

**Fig 4:**
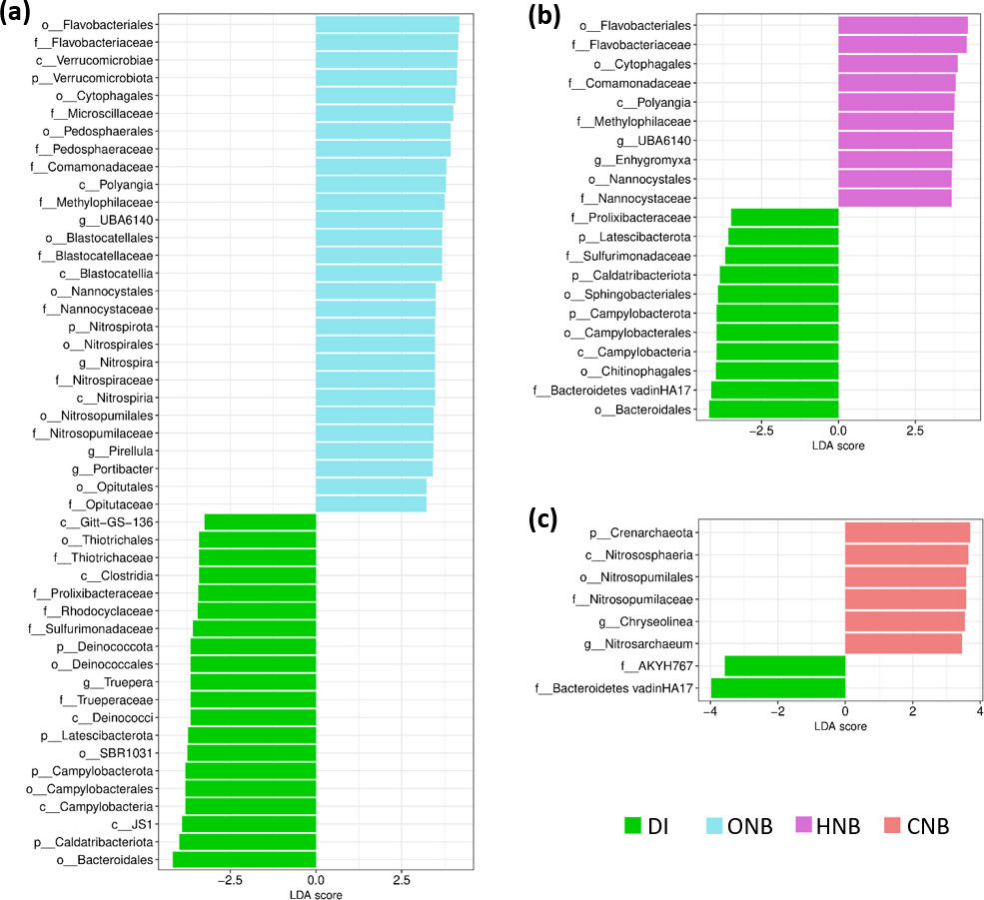
Differentially abundant bacterial taxa in soil samples under (**a**) ONB, (**b**) HNB, and (**c**) CNB treatments compared to the control (DI water). A positive LDA score indicates significant enrichment of the taxon compared to the control, while a negative LDA score indicates a reduction in relative abundance of the taxon compared to the control. The magnitude of the LDA score (shown on the x-axis in log scale) correlates with the magnitude of the change. Taxonomic ranks are included before taxon names (p: phylum, c: class, o: order, f: family, g: genus). Only taxa identified as significantly differentially abundant by both the Kruskal-Wallis test (*P* < 0.05) and Wilcoxon rank sum test (*P* < 0.1), and having relative abundances ≥0.05% of total reads, are shown.

The LEfSe method was used to identify bacterial taxa that were enriched by CNB, HNB, or ONB treatments relative to the DI control. Only data from week 3 and week 4 were included. The significant results were further filtered to retain only taxa that account for at least 0.05% of total reads. Figure 4 shows the taxa that were significantly enriched by each nanobubble treatment compared to the control and their LDA scores. A higher LDA score means the taxon showed a greater difference in abundance between the treated and control group. Overall, CNB-, HNB-, and ONB-treated groups were enriched in 6, 10, and 28 bacterial taxa compared to the DI control.

CNB treatment was associated with an increase in the abundances of the archaeal phylum *Crenarchaeota* and its component taxa, including the *Nitrosopumilaceae* family and *Nitrosarchaeum* genus, as well as the bacterial genus *Chryseolinea* (*Bacteroidota* phylum). *Nitrosopumilaceae* are adapted to low-oxygen environments, such as deep water (58), which likely enabled them to outcompete other taxa in the CNB-treated soil where DO was reduced to 2.0 mg·L^−1^. The microbial taxa enriched by CNBs compared to the control were fewer and less diverse than those enriched by HNBs and ONBs. This pattern is consistent with previous studies showing that elevated CO_2_ primarily influences soil microorganisms indirectly by stimulating plant growth and altering root exudation (59–61). For example, Wang et al. reported that elevated CO_2_ altered the diversity of the soybean rhizosphere microbiome but had no significant effects on the bulk soil microbiome (60). This may explain why the CNB-treated soil microbiome did not differ significantly from the control in this study. Moreover, CNB-induced reductions in soil pH and DO created acidic and microaerobic conditions that likely inhibited certain microbial taxa, potentially offsetting the nutrient mobilization and ROS-mediated effects of nanobubbles. Future studies that simultaneously assess plant physiological responses and soil microbial activity are thus needed to better elucidate the mechanisms underlying the plant growth benefits of CNBs.

HNB treatment was associated with an increase in the abundances of some *Bacteroidota* taxa, including *Flavobacteriales* and *Cytophagales*, some *Gammaproteobacteria* taxa, including *Comamonadaceae* and *Methylophilaceae*, and some *Myxococcota* taxa, including *Nannocystaceae*. Nearly all the taxa that were enriched by HNB treatment also increased in abundance following ONB treatment. The taxa promoted by both HNBs and ONBs, such as *Flavobacteriaceae*, *Comamonadaceae*, *Methylophilaceae*, and *Nannocystaceae*, likely benefited from the common properties of nanobubbles, such as soil nutrient mobilization (18). Since nanobubbles are negatively charged, they attract ions in the bulk solution to form an electric double layer on their surfaces (62, 63). This electric double layer can attract and adsorb the nutrient ions that are bound to soil particles, thus increasing the bioavailability of these nutrients. Xue et al. also reported that soil immersed in various nanobubble types, including CO_2_, O_2_, and H_2_ nanobubbles, showed increased conductivity, indicating higher levels of mobile ions (18). On the other hand, the absence of taxa that were enriched by HNBs but not ONBs was unexpected. Since hydrogen gas is known to reduce ROS accumulation while ONBs produce a high level of ROS (9, 64), HNBs were predicted to promote different groups of soil microbes than ONBs. Furthermore, HNBs significantly lowered soil DO and redox potential compared to the control, while ONBs significantly elevated soil DO, so the two treatments were expected to have opposite effects on the soil microbial communities. However, as indicated by the Adonis test, CCA, and LEfSe analyses, HNB- and ONB-treated soil microbiomes shared many similarities. One possible explanation is that the nutrient mobilization effects of HNBs and ONBs had stronger impacts on the soil microbiome than the changes in soil DO and redox potential, resulting in similar trends in HNB- and ONB-treated soil microbiomes. Furthermore, the highly reductive environment caused by HNBs was partially remedied by microbial activities, as evidenced by the increase in redox potential over time in HNB-treated soil. Since the difference in redox potential between HNB- and ONB-treated soils was attenuated, HNB treatment did not lead to a drastically different microbiome structure than ONB treatment.

Taxa that were exclusively enriched by ONBs included *Nitrospiraceae* (as well as its phylum *Nitrospirota*), *Blastocatellaceae* (*Acidobacteria* phylum), *Opitutaceae* and *Pedosphaeraceae* (*Verrucomicrobiota* phylum), *Pirellula* (*Planctomycetota* phylum), and *Portibacter* (*Bacteroidota* phylum). These taxa likely responded to the specific properties of oxygen nanobubbles, like elevated DO and ROS production (9). One of these taxa, *Nitrospirota*, also showed a positive correlation with ONB water treatment in a previous study (30).

Among the bacterial taxa enriched by both HNB and ONB treatments, many were known to play important roles in soil nutrient cycling, such as *Flavobacteriaceae*, *Comamonadaceae*, and *Nannocystaceae. Flavobacteriaceae* can break down many complex soil organic matters thanks to their ability to produce diverse extracellular enzymes, including cellulase, chitinase, peptidases, and glycoside hydrolases (65, 66). *Comamonadaceae* members possess genes related to carbon fixation, arsenic resistance, siderophore production (which improves plants’ access to iron), and sulfur cycling (67, 68). *Nannocystaceae* are halotolerant bacteria with the capabilities to lyse other bacteria, which may contribute to pathogen control, as well as degrade complex organic substances, such as agar, agarose, and casein (69). In addition, ONB treatment was associated with increases in the nitrifying families *Nitrosopumilaceae,* which convert ammonium to nitrite (58), and *Nitrospiraceae*, which oxidize nitrite to nitrate (70). One group that was only enriched by ONBs was *Blastocatellaceae*, whose members have been linked to the suppression of fungal pathogens in plants’ rhizosphere (71).

There were also overlaps between the taxa that decreased in abundance following CNB, HNB, and ONB treatments. Both CNB and HNB led to a decrease in the *Bacteroidetes vadinHA17* family, for example. Furthermore, both HNB and ONB treatments were associated with a reduction in the *Caldatribacteriota*, *Campylobacterota*, and *Latescibacterota* phyla, as well as the *Bacteroidales* order. Several taxa were selectively reduced following ONB treatment but not by other nanobubble types, including the *Clostridia* class, the *Gammaproteobacteria* families *Rhodocyclaceae* and *Thiotrichaceae*, and the *Truepera* genus.

### Nanobubble-specific modulation of soil microbial metabolic capacities

**Fig 5:**
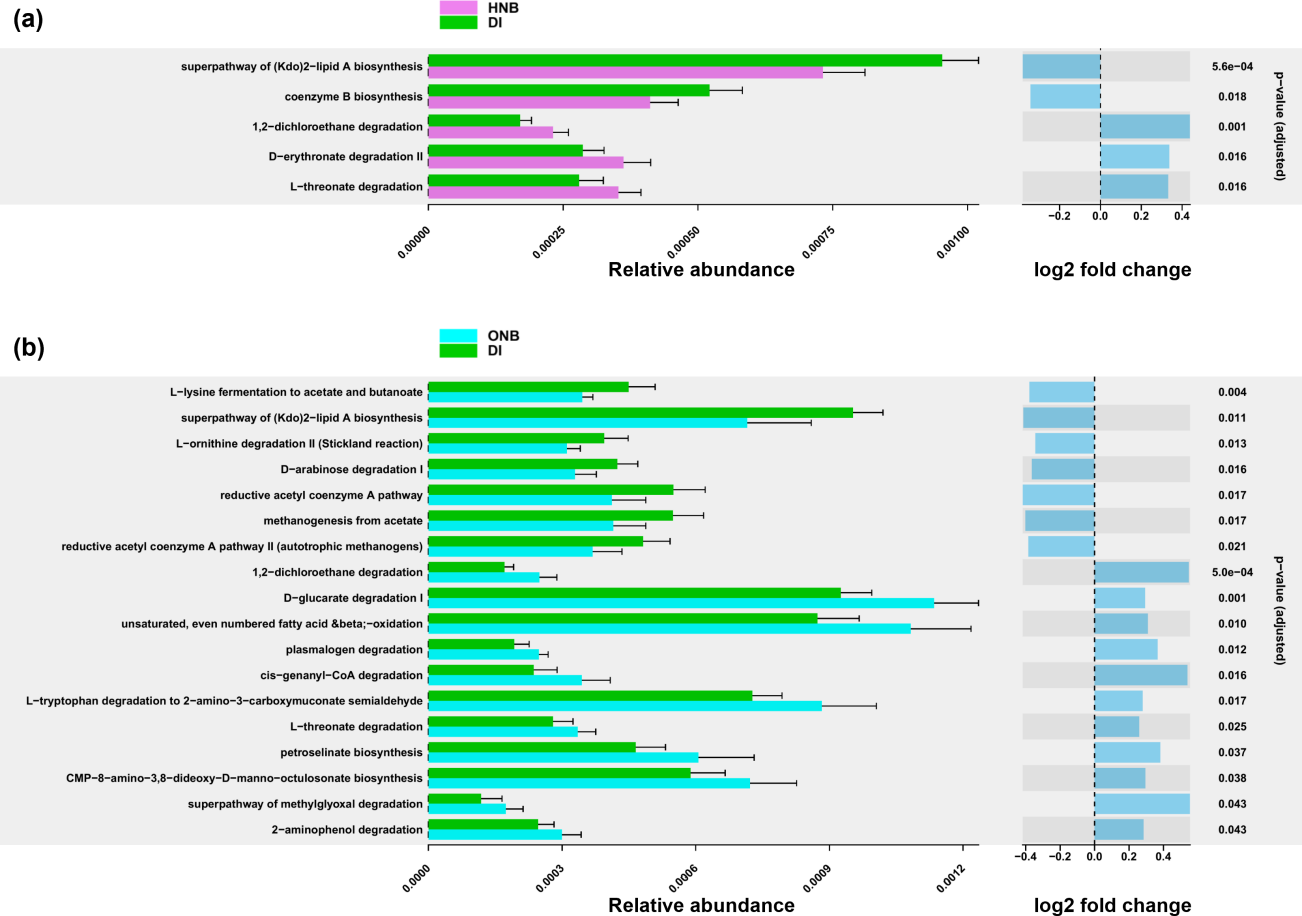
Differentially abundant metabolic pathways in soil samples under (**a**) HNB treatment and (**b**) ONB treatment compared to DI water control. Only pathways with adjusted *P* <0.05 and abundance changes >20% (log2 fold change > 0.26) are shown. No significantly altered pathways were detected in samples under CNB treatment.

Functional predictions based on the MetaCyc metabolic pathway were predicted using PICRUSt2, focusing on week 3 and week 4 and retaining pathways with relative abundances over 0.01% of total reads. CNB-treated samples had no differentially abundant metabolic pathways compared to the control. In contrast, HNB- and ONB-treated groups showed 13 and 50 differentially abundant metabolic pathways, respectively. Among these, five pathways in the HNB-treated group and 18 pathways for the ONB-treated group exhibited changes greater than 20% (log2 fold change > 0.26) (Fig. 5). For example, ONB significantly increased pathways associated with the degradation of various organic substances, including 1,2-dichloroethane, D-glucarate, cis-genanyl-CoA, L-tryptophan, L-threonate, methylglyoxal, and 2-aminophenol. This indicates that ONB irrigation expanded the substrate spectrum available to the soil bacteria, potentially leading to greater productivity and fertility. This finding agrees with the LEfSe result showing that ONBs enriched many bacterial taxa related to soil nutrient turnover (Fig. 4). In addition, the increases in the degradation pathways for 1,2-dichloroethane and 2-aminophenol, two industrial pollutants, indicate that the ONB-treated soil may be more effective in contaminant removal (72, 73). Conversely, ONBs reduced pathways related to L-lysine fermentation, (Kdo)2-lipid A biosynthesis, L-ornithine and D-arabinose degradation, the reductive acetyl coenzyme A pathway II of autotrophic methanogens, and acetoclastic methanogenesis. The suppression of methanogen-related pathways was likely due to the elevated DO levels in ONB-treated soil, consistent with previous findings that ONBs could inhibit methane production in rice paddies (28).

The functional changes under HNBs were fewer and narrower in scope. Notably, some pathways overlapped with those enriched or reduced under ONBs, such as increased degradation of 1,2-dichloroethane and L-threonate, as well as decreased (Kdo)2-lipid A biosynthesis. These results corroborate the LEfSe results, which showed that ONBs influenced a wider range of soil microorganisms than HNBs.

### Nanobubble-induced restructuring of soil microbial co-occurrence networks

**Fig 6:**
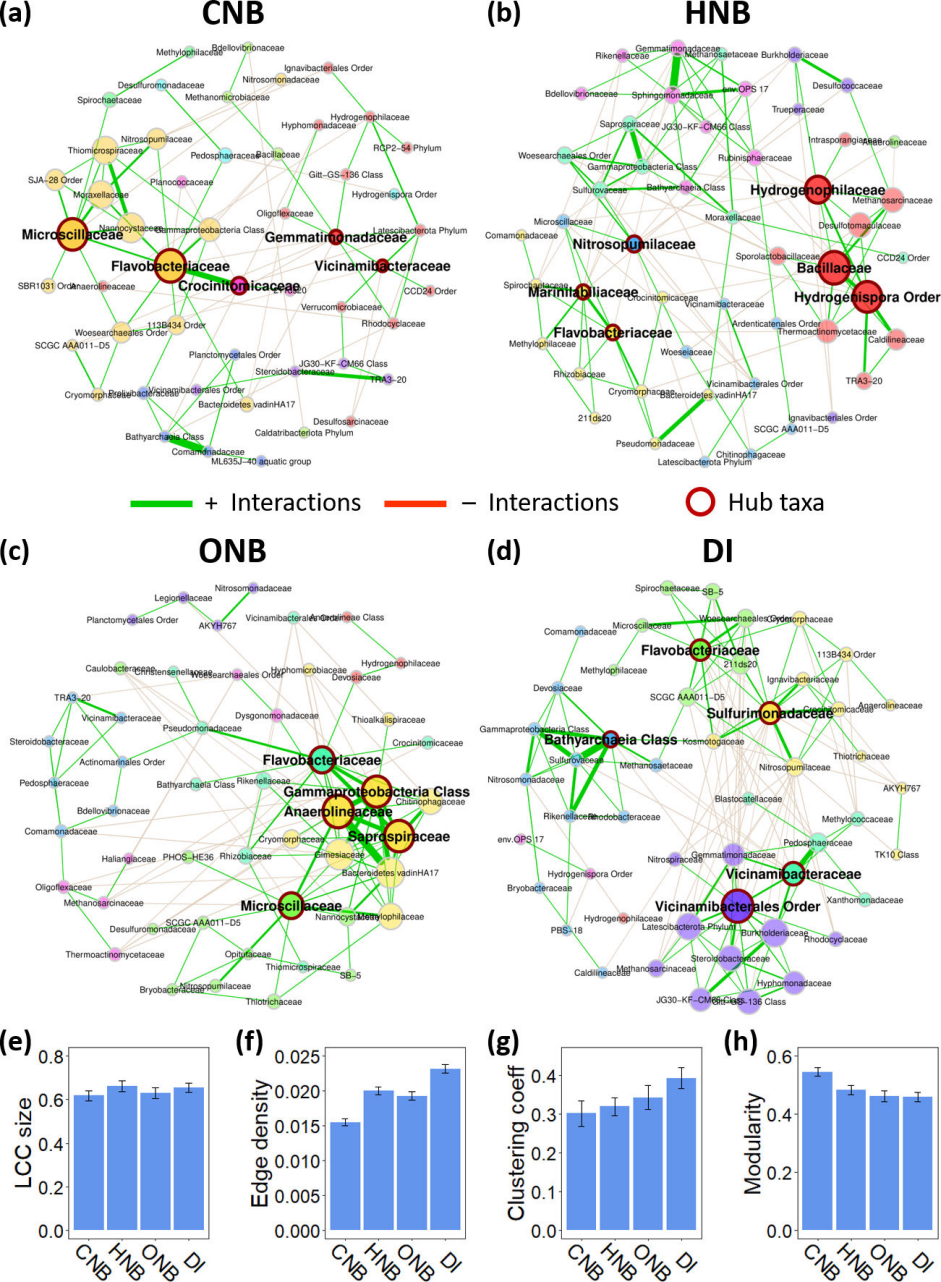
Co-occurrence networks showing the positive (green) and negative (orange) correlations between soil microbial families in samples irrigated with (**a**) CNB, (**b**) HNB, (**c**) ONB, and (**d**) DI water. Each network consists of 50 nodes representing the top 50 soil microbial taxa in terms of the number of significant connections. The node size represents eigenvector centrality (a measure of node importance), while node colors represent cluster membership. Nodes outlined in red are network hubs, which are potentially keystone taxa with central roles in the soil microbiome. Panels **e–h** summarize key topological properties of the networks: (**e**) largest connected component (LCC), (**f**) edge density, (**g**) clustering coefficient, and (**h**) modularity.

Figure 6 reveals the microbial co-occurrence networks under CNB, HNB, ONB, and DI water treatments. Hub taxa, referring to microbial families with disproportionately high centrality in the network, were identified based on both degree of connection and betweenness centrality. The hub families in CNB-treated microbiomes were *Microscillaceae, Flavobacteriaceae*, *Crocinitomicaceae*, *Gemmatimonadaceae,* and *Vicinamibacteraceae*. Three of these hubs (*Microscillaceae, Flavobacteriaceae*, and *Crocinitomicaceae*) belong to the *Bacteroidota*, a phylum specialized in polysaccharide degradation, phosphorus mineralization, and pathogen suppression (74, 75).

For HNB-treated microbiomes, the hubs were *Nitrosopumilaceae*, *Marinilabiliaceae*, *Flavobacteriaceae*, *Hydrogenophilaceae*, *Bacillaceae*, and unclassified families in the *Hydrogenispora* order. As previously discussed, HNBs significantly reduced the soil DO to 3.0 mg·L^−1^ and redox potential to below 0 mV compared to the control treatment. The lack of oxygen, the most energetically favorable terminal electron acceptor, typically promotes the use of alternative electron acceptors, such as nitrate, manganese, iron, sulfate, or carbon dioxide, favoring anaerobic and fermentative taxa while suppressing obligate aerobes (23). Contrary to this expectation, only *Hydrogenispora* and *Marinilabiliaceae* are anaerobic among the identified hubs (76, 77), whereas *Flavobacteriaceae* and *Bacillaceae* have mixed metabolic strategies (78, 79) and *Nitrosopumilaceae* and *Hydrogenophilaceae* are predominantly aerobic (80, 81). Similarly, the taxa enriched by HNBs were largely aerobic, including *Methylophilaceae* and *Nannocystaceae* (69, 82). A plausible explanation is that the nanobubble-mediated effects of HNBs, such as enhanced nutrient mobilization, may allow certain aerobic taxa to remain competitive despite the low redox conditions. In addition, one of the hub families was *Hydrogenophilaceae*, which are chemolithotrophs capable of oxidizing hydrogen and reduced sulfur compounds (80). The central role of *Hydrogenophilaceae* in the network suggests that this group contributed to the gradual increase in redox potential observed under HNB treatment and potentially provided niches for other aerobic taxa to thrive despite the highly reduced soil environment. Overall, these findings suggest that HNBs may elicit microbial responses distinct from those induced by hydrogen gas delivered via conventional methods.

For ONB-treated microbiomes, the hubs were *Flavobacteriaceae*, *Anaerolineaceae*, *Saprospiraceae*, *Microscillaceae*, and unclassified families in the *Gammaproteobacteria* class. Like the CNB-treated soil network, the hubs in ONB-treated soil were also dominated by *Bacteroidota* (*Flavobacteriaceae*, *Saprospiraceae*, and *Microscillaceae*). The DI control network contained five hubs, including *Flavobacteriaceae*, *Sulfurimonadaceae*, *Vicinamibacteraceae*, unclassified taxa in the *Vicinamibacterales* order, and unclassified taxa in the *Bathyarchaeia* class.

Notably, *Flavobacteriaceae* consistently emerged as a hub across all nanobubble treatment groups and control, suggesting its role as a keystone taxon, which are those that exert disproportionate effects on community structure and function regardless of their abundances (83). The significantly lower relative abundance of *Flavobacteriaceae* in control samples compared to HNB- and ONB-treated samples did not diminish the importance of this family to the control soil network, demonstrating that its centrality was due to metabolic versatility rather than population size. By promoting the keystone taxon *Flavobacteriaceae*, HNBs and ONBs could indirectly enhance the activity of many other connected taxa, thus enhancing the overall productivity of the soil microbiome. ONBs also significantly enriched *Microscillaceae*, a hub taxon in both CNB- and ONB-treated soil. Not much is known about the metabolism of *Microscillaceae*, but their participation in gelatin degradation and sulfur cycling (84, 85) supports their ecological importance. Overall, the detection of keystone microbial taxa responsive to nanobubble treatments demonstrates a new mechanism by which nanobubbles can modulate the soil microbiome to promote soil and plant health.

Network topological properties are summarized in Fig. 6e through h. Edge density is the ratio of existing interactions to the maximum number of possible interactions; clustering coefficient measures how closely the nodes are connected; and modularity is the tendency of the network to be divided into separate cliques. CNB-treated microbiomes showed the lowest edge density and clustering coefficient, but the highest modularity among all treatment and control groups. These characteristics indicate a sparse and highly compartmentalized network, where different clusters adapted to distinct environmental niches but did not cooperate much. These niches might have resulted from the unique impacts of CNBs on soil chemistry, including lowered pH, increased cation solubility, and enhanced phosphate release (18). In contrast, HNB- and ONB-treated microbiomes exhibited denser and more clustered networks, demonstrating stronger microbial cooperation and higher functional redundancy. While the control network had higher edge density and clustering coefficients than the HNB- and ONB-treated networks, two out of five hubs in the control network were unclassified at the family level, making comparisons to the nanobubble-treated network inconclusive.

### Conclusion

This study provides the first in-depth investigation into how nanobubble-enriched irrigation water with different gaseous compositions modulates soil chemical properties and microbial communities. The treatments created distinct soil environments, notably altering dissolved oxygen, pH, and redox potential. Integrated analyses of microbial diversity, taxonomic shifts, metabolic functions, and network topology demonstrated that ONBs and HNBs exerted the most significant and beneficial impacts on the soil microbiome. Although alpha diversity remained largely unchanged, ONB- and HNB-treated soils were enriched in microbial taxa crucial for nutrient cycling, organic matter turnover, and pathogen suppression, particularly *Flavobacteriaceae*, *Comamonadaceae*, *Nannocystaceae*, and *Blastocatellaceae*. These microbial shifts were accompanied by enhanced metabolic pathways related to pollutant degradation and organic substrate utilization. Network analysis further identified *Flavobacteriaceae* as a keystone taxon, suggesting that nanobubbles not only reshape microbial communities but also strengthen their ecological resilience and functional capacity. In contrast, CNBs had limited effects on microbial composition and function beyond promoting *Nitrosopumilaceae*, likely due to adverse impacts on soil pH and oxygen availability. The enrichment of beneficial and functionally critical microbial taxa under ONB and HNB water irrigation highlights the potential of nanobubble technology as a microbiome engineering tool for sustainable agriculture. Moving forward, research should explore the optimization of gas composition, delivery regimes, and compatibility with diverse crops and soil systems. This work lays the foundation for scalable, low-input, and environmentally friendly irrigation strategies that enhance soil health, boost crop productivity, and increase agroecosystem resilience.

## Data Availability

Sequence data have been submitted to the NCBI Sequence Read Archive under the BioProject accession number PRJNA1195671.

## SUPPLEMENTAL MATERIAL

Supplemental material aem.02173-25-s0001.docx Text S1 and S2, Fig. S1 to S4, and Tables S1 to S3.

ASM does not own the copyrights to Supplemental Material that may be linked to, or accessed through, an article. The authors have granted ASM a non-exclusive, world-wide license to publish the Supplemental Material files. Please contact the corresponding author directly for reuse.
