# Supplementary material for: Soil chemistry and microbiome modulation through water irrigation containing oxygen, hydrogen, and carbon dioxide nanobubbles
Source: Appl Environ Microbiol. 2026 Mar 30;92(4):e02173-25. doi: 10.1128/aem.02173-25 (PMC13101497; doi:10.1128/aem.02173-25)
Supplement: Supplemental material — Text S1 and S2, Fig. S1 to S4, and Tables S1 to S3. [file aem.02173-25-s0001.docx]

**Supporting Information**

**For**

**Soil Chemistry and Microbiome Modulation through Water Irrigation Containing Oxygen, Hydrogen, and Carbon Dioxide Nanobubbles**

Nguyen Nhat Thu Le^1,2^, Shan Xue^2,3^, Hui Mu^2,4^, Jianfeng Wu^1^, Chuanwu Xi^1^, Taha Marhaba^2^, Wen Zhang^2^*

^1^Department of Environmental Health Sciences, University of Michigan School of Public Health, Ann Arbor, MI 48109, USA

^2^Department of Civil and Environmental Engineering, New Jersey Institute of Technology, Newark, NJ 07102, USA

^3^PureNanoTech Inc., 239 New Rd A 104, Parsippany, NJ 07054, USA

^4^School of Water Conservancy and Environment, University of Jinan, Jinan, 250022, China

*Corresponding author: Wen Zhang, E-mail: [wen.zhang@njit.edu](mailto:wen.zhang@njit.edu)

# Text S1. Bioinformatics and microbiome analysis details

## S1-1. Sequence quality control and normalization

A total of 609,932 raw sequences were obtained for 80 irrigated soil samples and five untreated soil samples. Data pre-processing and quality control were done using DADA2 (v. 1.32.0) in R v. 4.4.1.^1^ Specifically, forward reads were trimmed to 240 bp and reverse reads to 230 bp, followed by filtering to remove reads with ambiguous bases and more than 2 expected errors. Pair-end reads were merged, and sequences shorter than 250 bp or longer than 256 bp were discarded. Chimeras sequences were removed, and the remaining 390,775 high-quality sequences were aligned to the SILVA database (v. 138.1). The resulting amplicon sequence variant (ASV) table was further filtered to remove ASVs with unclassified phyla and ASVs with fewer than 5 total counts. One sample with fewer than 1000 reads after quality control was excluded from downstream analyses. The ASV table was rarefied to 2120 reads per sample spanning 2646 ASVs.

## S1-2. Changes in microbial diversity and community composition

Alpha diversity (observed richness and Shannon index) was calculated by the phyloseq package.^2^ To evaluate differences in microbial community structure among treatment groups, non-metric multidimensional scaling (NMDS) and pairwise Adonis test based on Bray-Curtis dissimilarity were performed using the R packages vegan and pairwiseAdonis. Canonical correspondence analysis (CCA) was performed using R package ggvegan to investigate the effect of increasing duration of nanobubble exposure on microbial community structures of soil and rhizosphere samples.

The linear discriminant analysis effect size (LEfSe) method was used to analyze the effects of different nanobubble treatments on specific bacterial taxa at the phylum, class, order, family, and genus levels.^3^ This analysis was implemented using the MicrobiomeMarker package.^4^ Bacterial taxa with statistically significant differences in abundances across treatment groups were identified as microbiome markers using the Kruskal-Wallis test (*p* < 0.05). Then, samples were divided into subgroups by sampling time, and pair-wise Wilcoxon rank sum tests were performed within each subgroup. Only taxa with differential abundances in each subgroup (*p* < 0.1) as well as the Kruskal-Wallis test were retained as biomarkers.

## S1-3. Predicted functional profile of the microbiome

The Phylogenetic Investigation of Communities by Reconstruction of Unobserved States (PICRUSt2) v2.3.2 was used to predict the functional profiles of the soil microbiome samples.^5^ The SEPP method was used for read alignment.^6^ Metabolic pathway abundances were predicted using the MetaCyc database and filtered to remove pathways that were no more than 0.01% in relative abundance. Then, differentially abundant pathways between the nanobubbles treatment groups and the control were identified using the Linear Models for Differential Abundance Analysis (LinDA) method (*p* < 0.05 after Benjamini-Hochberg adjustment), as implemented in the ggpicrust2 R package.^7^

## S1-4. Microbial network analysis

The microbial co-occurrence network of each treatment group was analyzed using the NetCoMi package.^8^ The ASV table was agglomerated at the family level and filtered to retain only families with relative abundance ≥ 0.1%. Significant interactions (*p* < 0.05) were inferred using the compositionally aware SparCC method.^9^ The networks were iterated 100 times to obtain the means and standard deviations of network parameters such as average size of the largest connected component, clustering coefficient, edge density, and modularity. The hubs were defined as the taxa in the 95^th^ percentile for both degree and betweenness centralities in order to identify taxa with high connectivity both within and between clusters.


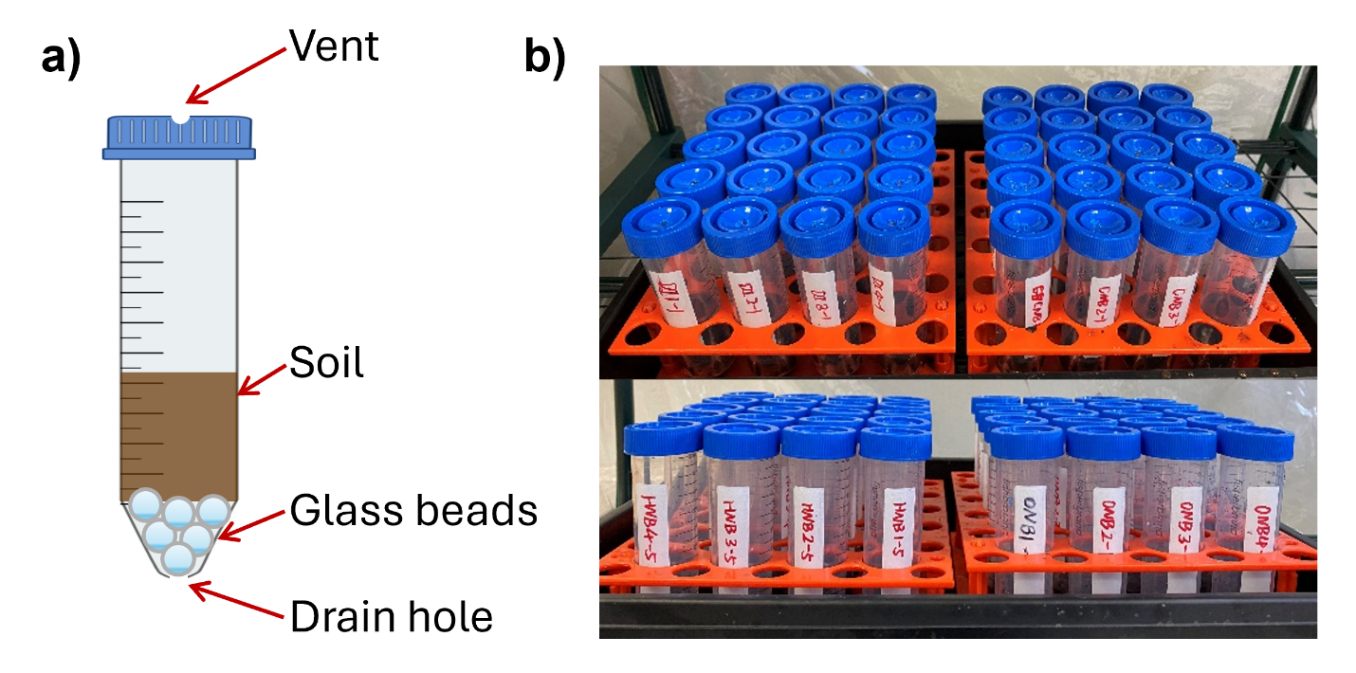


# Figure S1. Experimental setup: (a) soil column structure; (b) soil samples transferred to preservative solution before shipping to the University of Michigan for DNA sequencing.

# Text S2. Nernst equation and the effect of pH on redox potential

A generic oxidation-reduction half-reaction can be represented as follows:

$a\text{Oxidized}+m\text{H}^{\text{+}}+n\text{e}^{\text{-}}\rightleftharpoons b\text{Reduced}$ (1)

The redox potential (*E*) of this half-reaction is given by the Nernst equation below:

$E=E^{\circ}-\frac{RT}{nF}\ln\left(\frac{\left[\text{Reduced}\right]^{b}}{\left[\text{Oxidized}\right]^{a}\left[\text{H}^{\text{+}}\right]^{m}}\right)$ (2)

where

*E*: Redox potential of the half reaction at the given temperature and reagent concentrations (V)

*E*°: Redox potential of the half reaction under standard conditions (V)

*R*: Universal gas constant (8.314 J·mol^−1^·K^−1^)

*T*: Temperature (K)

*F*: Faraday constant (96,485 C·mol^−1^)

By converting to base-10 logarithm and substituting $\text{pH}=-\log\left[\text{H}^{\text{+}}\right]$, we get the following equation at *T* = 298.15 K or 25°C:

$E=E^{\circ}-\frac{0.0592}{n}\left[\log\left(\frac{\left[\text{Reduced}\right]^{b}}{\left[\text{Oxidized}\right]^{a}}\right)+m\left(\text{pH}\right)\right]$ (3)

Equation (3) shows that without significant changes in the concentrations of oxidized and reduced species, a reduction in pH would lead to an increase in solution redox potential. To further verify this, we can examine the redox reactions specific to the tested nanobubble suspensions. The redox potentials of suspensions of oxygen nanobubbles (ONBs) and hydrogen nanobubbles (HNBs) are influenced by many potential half-reactions, some of which are shown below:

$O_{2(g)}+4e^{-}+4H^{+}\to2H_{2}O_{(l)}$ $E^{\circ}$= 1.23 V (4)

$2H_{2}O_{(l)}+2e^{-}\to H_{2(g)}+2OH^{-}_{(aq)}$ $E^{\circ}$= −0.83 V (5)

$\bullet OH_{(aq)}\text{ }+e^{-}+H^{+}\to H_{2}O_{(l)}$ $E^{\circ}$= 2.72 V (6)

Using the Nernst Equation, we obtained the E-pH relation for the above three reactions (assuming a partial pressure of 1 atm for gases inside nanobubbles):

For reaction (4) at pH = 6.5:

$$E=E^{\circ}+\left(\frac{0.0592}{4}\right)\left[\log\left(P_{O_{2}}\right)-4pH\right]=0.85\text{ V}$$

For reaction (5) at pH = 6.5:

$$E=E^{\circ}-\left(\frac{0.0592}{2}\right)\log\left(P_{H_{2}}\right)+0.0592\left(14-pH\right)=-0.39\text{ V}$$

For reaction (6) at pH = 6.5:

$$E=E^{\circ}-0.0592\left(pH-\log\left[\bullet OH\right]\right)$$

The redox potential of a carbon dioxide nanobubble suspension is also influenced by many half-reactions, some of which are listed below:

$CO_{2(g)}+2e^{-}+2H^{+}\to HCOOH_{(l)}$ $E^{\circ}$= −0.61 V (7)

$CO_{2(g)}+2e^{-}+2H^{+}\to CO_{(g)}+H_{2}O_{(l)}\text{ }$ $E^{\circ}$= −0.53 V (8)

$CO_{2(g)}+4e^{-}+4H^{+}\to HCHO_{(l)}+H_{2}O_{(l)}\text{ }$ $E^{\circ}$= −0.48 V (9)

$CO_{2(g)}+6e^{-}+6H^{+}\to CH_{3}OH_{(l)}+H_{2}O_{(l)}$ $E^{\circ}$= −0.38 V (10)

$E^{\circ}$= −0.24 V (11)

$E^{\circ}$= 0.06 V (12)

Using the Nernst Equation, we obtained the E-pH relation for reaction (7) at pH = 4.4 and
$P_{CO_{2}}$= 1 atm for pure gas inside nanobubbles:

$$E=E^{\circ}+\left(\frac{0.0592}{2}\right)\left[\log\left(P_{CO_{2}}\right)-2pH\right]=-0.87\text{ V}$$

The other half-reactions follow the same relationship between E and pH. We can thus conclude that in suspensions of nanobubbles, a decrease in pH leads to an increase in redox potential and vice versa.

# Table S1. Results from Kruskal-Wallis tests and Conover-Iman post hoc tests comparing the dissolved oxygen (DO) levels, pH, and redox potentials of soil samples exposed to CNB, HNB, ONB, or DI (control) at each sampling time (Week 1 to Week 4). Bold numbers indicate statistically significant differences (*p* < 0.05 for Kruskal-Wallis test and adjusted *p* < 0.025 for post hoc test).

|  | *Week 1* | *Week 2* | *Week 3* | *Week 4* |
| --- | --- | --- | --- | --- |
| ***DO level*** | | | | |
| *Kruskal-Wallis* | **3.38E-05** | **3.22E-05** | **2.07E-05** | **2.54E-05** |
| CNB vs. DI | **< 0.0001** | **< 0.0001** | **< 0.0001** | **< 0.0001** |
| HNB vs. DI | **< 0.0001** | **< 0.0001** | **< 0.0001** | **< 0.0001** |
| ONB vs. DI | **0.0002** | **0.0002** | **< 0.0001** | **0.0001** |
| CNB vs. HNB | 0.0333 | **0.0224** | **< 0.0001** | **0.0030** |
| CNB vs. ONB | **< 0.0001** | **< 0.0001** | **< 0.0001** | **< 0.0001** |
| HNB vs. ONB | **< 0.0001** | **< 0.0001** | **< 0.0001** | **< 0.0001** |
| ***pH*** | | | | |
| *Kruskal-Wallis* | **0.0034** | **0.0004** | **0.0011** | **0.0011** |
| CNB vs. DI | **0.0029** | **0.001** | **0.0006** | **0.0006** |
| HNB vs. DI | 0.3979 | 0.0672 | 0.2209 | 0.2210 |
| ONB vs. DI | 0.1318 | **0.0085** | 0.1307 | 0.1308 |
| CNB vs. HNB | **0.0023** | **< 0.0001** | **0.0001** | **< 0.0001** |
| CNB vs. ONB | **0.0003** | **< 0.0001** | **0.0001** | **0.0001** |
| HNB vs. ONB | 0.1609 | 0.143 | 0.3168 | 0.3169 |
| ***Redox potential*** | | | | |
| *Kruskal-Wallis* | **0.0001** | **0.0003** | **3.63E-05** | **0.0002** |
| CNB vs. DI | **0.0001** | **0.004** | **< 0.0001** | **0.0006** |
| HNB vs. DI | **0.0001** | **0.0008** | **0.0001** | **0.0004** |
| ONB vs. DI | 0.2225 | 0.0834 | **0.0019** | 0.1018 |
| CNB vs. HNB | **< 0.0001** | **< 0.0001** | **< 0.0001** | **< 0.0001** |
| CNB vs. ONB | **0.0007** | 0.0695 | **0.0004** | **0.0104** |
| HNB vs. ONB | **< 0.0001** | **< 0.0001** | **< 0.0001** | **< 0.0001** |


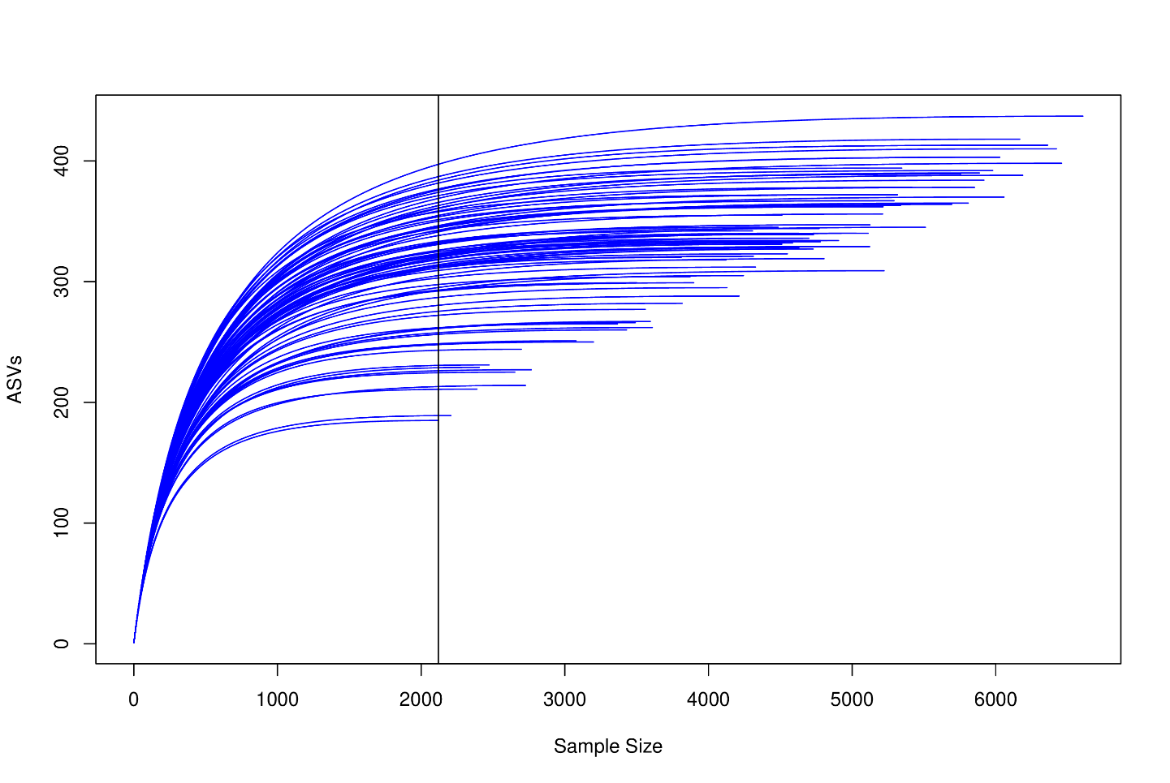


# Figure S2. Rarefaction curves showing that the number of ASVs is close to plateauing at the selected sampling depth (2120 reads per sample).

**
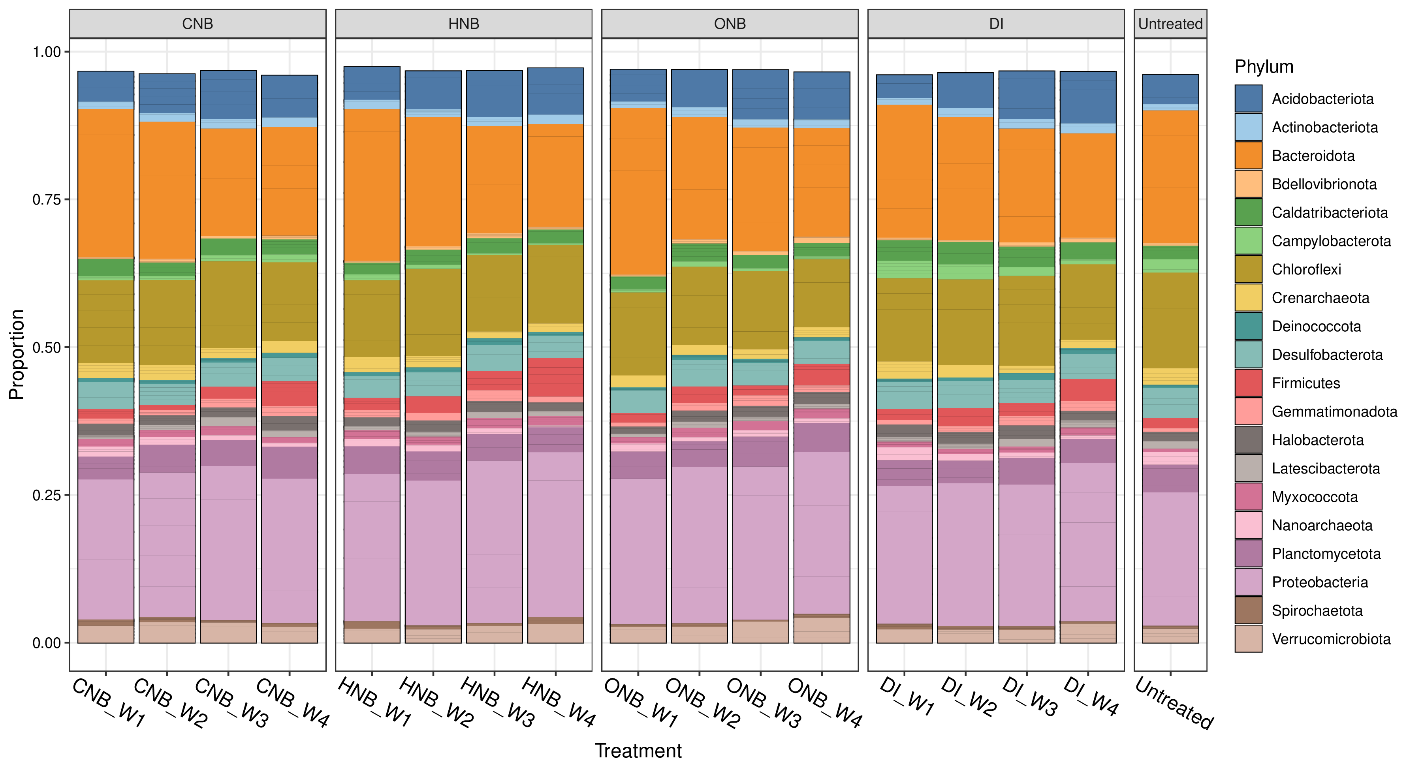
**

# Figure S3. Relative abundance of dominant phyla in soil microbiomes treated with CNB, HNB, ONB, as well as DI water and the untreated bulk soil. Samples were collected every week from Week 1 (W1) to Week 4 (W4). Only the top 20 phyla are shown.

**
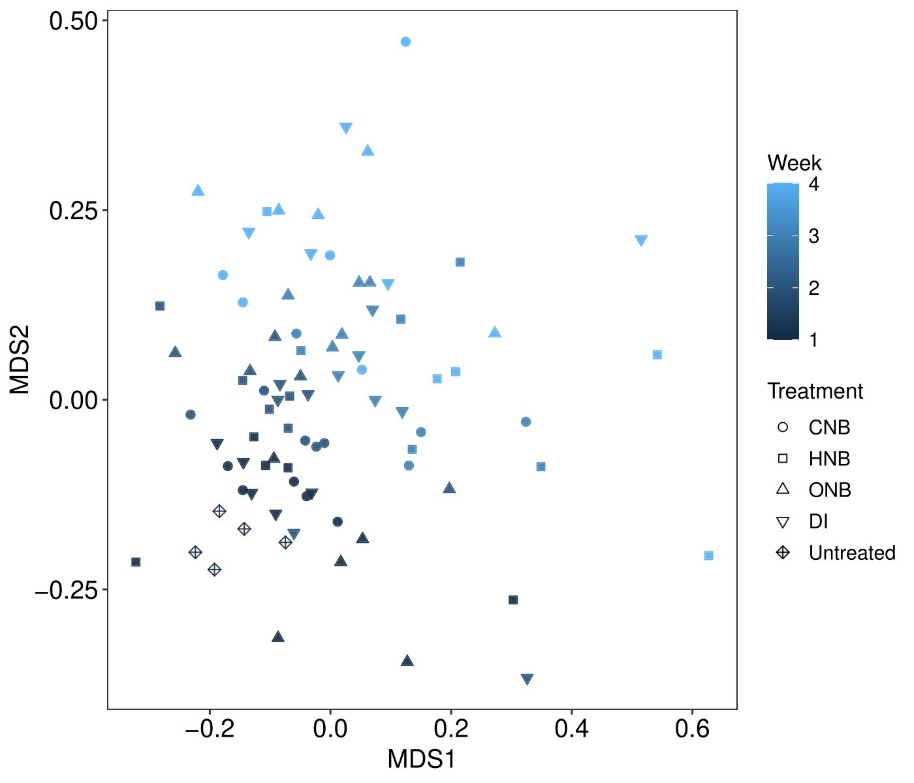
**

# Figure S4. Non-metric multidimensional scaling (NMDS) plots of soil microbial community structures following exposure to CNB, HNB, ONB, or DI (control), as well as untreated bulk soil. Samples were collected every week for 4 weeks.

# Table S2. Results from pairwise Adonis tests comparing the microbial community structures of soil samples with different treatment durations (1 week to 4 weeks) in each treatment group (CNB, HNB, ONB, DI water). Bold numbers indicate statistically significant differences (*p*-values < 0.05).

|  | *CNB* | *HNB* | *ONB* | *DI* |
| --- | --- | --- | --- | --- |
| Week 1 vs. Week 2 | **0.008** | 0.16 | **0.007** | **0.010** |
| Week 1 vs. Week 3 | **0.010** | **0.018** | **0.008** | **0.008** |
| Week 1 vs. Week 4 | **0.009** | **0.009** | **0.009** | **0.007** |
| Week 2 vs. Week 3 | **0.008** | **0.018** | **0.021** | **0.008** |
| Week 2 vs. Week 4 | **0.008** | **0.009** | **0.008** | **0.008** |
| Week 3 vs. Week 4 | 0.057 | 0.055 | **0.047** | 0.10 |

# Table S3. Results from pairwise Adonis tests comparing the microbial community structures of soil samples exposed to CNB, HNB, ONB, or DI (control) at each sampling time (Week 1 to Week 4). Bold numbers indicate statistically significant differences (*p*-values < 0.05).

|  | *Week 1* | *Week 2* | *Week 3* | *Week 4* |
| --- | --- | --- | --- | --- |
| CNB vs. DI | **0.007** | **0.009** | 0.054 | 0.073 |
| HNB vs. DI | **0.009** | **0.008** | **0.008** | **0.016** |
| ONB vs. DI | **0.009** | **0.010** | **0.008** | **0.008** |
| CNB vs. HNB | 0.15 | **0.029** | 0.25 | **0.026** |
| CNB vs. ONB | **0.007** | **0.005** | **0.008** | **0.009** |
| HNB vs. ONB | 0.21 | 0.37 | **0.023** | 0.35 |

# References

1. Callahan, B. J.; McMurdie, P. J.; Rosen, M. J.; Han, A. W.; Johnson, A. J. A.; Holmes, S. P., DADA2: High-resolution sample inference from Illumina amplicon data. *Nature Methods* **2016,** *13*, 581-583.

2. McMurdie, P. J.; Holmes, S., phyloseq: an R package for reproducible interactive analysis and graphics of microbiome census data. *PloS One* **2013,** *8*, e61217.

3. Segata, N.; Izard, J.; Waldron, L.; Gevers, D.; Miropolsky, L.; Garrett, W. S.; Huttenhower, C., Metagenomic biomarker discovery and explanation. *Genome biology* **2011,** *12*, 1-18.

4. Cao, Y.; Dong, Q.; Wang, D.; Zhang, P.; Liu, Y.; Niu, C., microbiomeMarker: an R/Bioconductor package for microbiome marker identification and visualization. *Bioinformatics* **2022,** *38*, 4027-4029.

5. Douglas, G. M.; Maffei, V. J.; Zaneveld, J. R.; Yurgel, S. N.; Brown, J. R.; Taylor, C. M.; Huttenhower, C.; Langille, M. G. I., PICRUSt2 for prediction of metagenome functions. *Nature Biotechnology* **2020,** *38*, 685-688.

6. Mirarab, S.; Nguyen, N.; Warnow, T. In *SEPP: SATé-Enabled Phylogenetic Placement*, Pacific Symposium on Biocomputing, 2011; Altman, R. B., et al., Eds. 2012; pp 247-258.

7. Yang, C.; Mai, J.; Cao, X.; Burberry, A.; Cominelli, F.; Zhang, L., ggpicrust2: an R package for PICRUSt2 predicted functional profile analysis and visualization. *Bioinformatics* **2023,** *39*, btad470.

8. Peschel, S.; Müller, C. L.; Von Mutius, E.; Boulesteix, A. L.; Depner, M., NetCoMi: network construction and comparison for microbiome data in R. *Briefings in Bioinformatics* **2021,** *22*, bbaa290.

9. Friedman, J.; Alm, E. J., Inferring correlation networks from genomic survey data. *PLOS Computational Biology* **2012,** *8*, e1002687.
